# Time to death and predictors of mortality among early neonates admitted to neonatal intensive care unit of Addis Ababa public Hospitals, Ethiopia: Institutional-based prospective cohort study

**DOI:** 10.1371/journal.pone.0302665

**Published:** 2024-06-06

**Authors:** Erean Shigign Malka, Tarekegn Solomon, Dejene Hailu Kassa, Besfat Berihun Erega, Derara Girma Tufa

**Affiliations:** 1 School of Public Health, College of Medicine and Health Sciences, Salale University, Salale, Ethiopia; 2 School of Public Health, College of Medicine and Health Sciences, Hawassa University, Hawassa, Ethiopia; 3 School of Midwifery, College of Health Sciences, Debre Tabor University, Debre Tabor, Ethiopia; UiA: Universitetet i Agder, NORWAY

## Abstract

**Introduction:**

The largest risk of child mortality occurs within the first week after birth. Early neonatal mortality remains a global public health concern, especially in sub-Saharan African countries. More than 75% of neonatal death occurs within the first seven days of birth, but there are limited prospective follow- up studies to determine time to death, incidence and predictors of death in Ethiopia particularly in the study area. The study aimed to determine incidence and predictors of early neonatal mortality among neonates admitted to the neonatal intensive care unit of Addis Ababa public hospitals, Ethiopia 2021.

**Methods:**

Institutional prospective cohort study was conducted in four public hospitals found in Addis Ababa City, Ethiopia from June 7^th^, 2021 to July 13^th^, 2021. All early neonates consecutively admitted to the corresponding neonatal intensive care unit of selected hospitals were included in the study and followed until 7 days-old. Data were coded, cleaned, edited, and entered into Epi data version 3.1 and then exported to STATA software version 14.0 for analysis. The Kaplan Meier survival curve with log- rank test was used to compare survival time between groups. Moreover, both bi-variable and multivariable Cox proportional hazard regression model was used to identify the predictors of early neonatal mortality. All variables having P-value ≤0.2 in the bi-variable analysis model were further fitted to the multivariable model. The assumption of the model was checked graphically and using a global test. The goodness of fit of the model was performed using the Cox-Snell residual test and it was adequate.

**Results:**

A total of 391 early neonates with their mothers were involved in this study. The incidence rate among admitted early neonates was 33.25 per 1000 neonate day’s observation [95% confidence interval (CI): 26.22, 42.17]. Being preterm birth [adjusted hazard ratio (AHR): 6.0 (95% CI 2.02, 17.50)], having low fifth minute Apgar score [AHR: 3.93 (95% CI; 1.5, 6.77)], low temperatures [AHR: 2.67 (95%CI; 1.41, 5.02)] and, resuscitating of early neonate [AHR: 2.80 (95% CI; 1.51,5.10)] were associated with increased hazard of early neonatal death. However, early neonatal crying at birth [AHR: 0.48 (95%CI; 0.26, 0.87)] was associated with reduced hazard of death.

**Conclusions:**

Early neonatal mortality is high in Addis Ababa public Hospitals. Preterm birth, low five-minute Apgar score, hypothermia and crying at birth were found to be independent predictors of early neonatal death. Good care and attention to neonate with low Apgar scores, premature, and hypothermic neonates.

## Introduction

Neonates are young infants found in the age category of birth to 28 days of life. Neonatal mortality is defined as the probability of dying of a live born infant in the first 28 days of life. Neonatal death can be further classified into: (a) early neonatal deaths which occur from birth day to the seventh day of life (0 to <7 days), and (b) late neonatal deaths which occur after the seventh day but before 28 completed days of life (≥7 to <28 days. The time elapse from birth to the seventh day of life is the most vulnerable time for the neonate because major physiologic adjustments are needed for extra-uterine life [1].

Survival of a neonate during the first week of age is determined not only by the stresses of intrauterine life but also by the birth process as well as by the adjustment to a new environment, nutrition, and infection. So, early neonatal period, birth to 7 days of life, is the most critical period of life.

Although the probability of survival has increased for all age groups since 2000, it was not similar progress for all childhood ages. Globally, there has been a substantial decline in infant and under-five mortality in the past decades. The largest improvements in survival for children under 5 years of age occurred among children aged 1−4 years. However, neonatal mortality remained relatively unchanged especially in developing countries, including in Ethiopia [2, 3].

According to United Nations Inter-Agency Group for Child Mortality Estimation 2020 report, Children face the highest risk of dying in their first month of life at an average global rate of 17 deaths per 1,000 live births in 2019, down by 52 percent from 38 deaths per 1,000 in 1990. In another word, 2.4 million children died during the first month of life in 2019, which is approximated to 6,700 neonatal deaths every day [4].

The magnitude of child deaths is not evenly distributed across all regions [4]. Most of these newborn deaths (99%) take place in low-income countries [3, 5]. Two regions account for almost 80% of all neonatal deaths in 2019; Sub-Saharan Africa accounted for 42% of all such deaths and Central and Southern Asia accounted for 37% [4]. In Ethiopia, neonatal mortality rates account for 41% of under-five deaths. It had been continuously declined from 39 deaths per 1000 live birth in 2005 to 29 deaths per 1000 live birth in 2016. However, the neonatal mortality rate has been increased to 30 deaths per 1,000 live births in 2019. Moreover, studies conducted in Sub-Saharan-Africa and some parts of Ethiopia show high magnitudes of neonatal mortality [6–9].

The common causes of neonatal mortality and morbidity are intrapartum-related complications, maternal related factors and sepsis. Preterm birth (40.8%) and intrapartum complications (27.0%) accounted for most early neonatal deaths while infections caused nearly half of late neonatal deaths [2, 4, 8, 10, 11]. About three fourth of the neonatal deaths in low and middle income countries can be prevented through effective schemes with existing simple and low cost tools, like Antenatal care, kangaroo mother care for preterm babies, early breast feed initiation, antibiotics for pneumonia and sepsis, newborn resuscitation, skin-to-skin contact, clean water, use of disinfectant, and good nutrition along with access to well-trained healthcare providers [3, 12, 13].

Efforts have been made globally as well as nationally to prevent the death of neonate. Globally, it was included in and remained the ‘unfinished’ agenda’ of Millennium Development Goals which was extended to Sustainable Development Goals. Accordingly, target two of Sustainable Development Goals three is planned to end preventable deaths of newborns in all countries aiming to reduce neonatal mortality to at least as low as 12 per 1000 live births by 2030 [14]. Ethiopia had further planned to reduce the neonatal mortality rate from 28 deaths per 1000 live birth in 2015/16 to 10 deaths by 2019/2020. To do so, plans and strategies like Integrated Management of Newborn and Childhood Illness strategy, Kangaroo Mother Care and Health Sector Development Plan had been formulated [15–17].

Despite these policies and intervention initiatives, currently, Ethiopia is among the countries with a high number of newborn deaths in Africa and globally [4]. Moreover, national and international shreds of evidence indicate that from neonatal death, one-third of neonatal mortality occurs during the first 24 hours of birth and about 75% of NM occurs during the first week of life [4, 8, 12]. According to Ethiopian demographic health survey 2016 report, perinatal mortality rate (the sum of the number of stillbirths and early neonatal deaths) in Ethiopia was 33 deaths per 1000 pregnancies [7]. But there are limitations of evidence on time to death, incidence and predictors of early neonatal life in Ethiopia particularly in study area. Besides, prospective cohort study is rarely conducted on early neonatal death.

Therefore, representative and methodologically sound investigations need to be done by far, more substantially in the Addis Ababa public Hospitals. Perceiving this, the study was conducted to assess incidence and predictors of early neonatal mortalities among neonates admitted to neonatal intensive care unit of Addis Ababa public Hospitals, Ethiopia, 2021.

## Methods

### Study setting

The study was conducted in public hospitals found in Addis Ababa, the capital city of Ethiopia. It is situated in the geographic center of the country on a well-watered plateau surrounded by hills and mountains. According to a population projection value for 2020, the city has an estimated population of 4.8 million people. It covers an area of 527 square kilometers and has 11 sub cities [18].

In Addis Ababa City, there are twelve public Hospitals; from which six are found under the administration of Addis Ababa City Health Bureau, one by Addis Ababa University and the remaining five are governed by Ethiopian Federal Ministry of Health. Except for Amanuel Mental Hospital, each of these Hospitals has its own neonatal intensive care unit. The study was carried out in four randomly selected public Hospitals that have their own neonatal intensive care unit (NICU). Those selected were Yekatit 12 Medical College Hospital, Gandhi Memorial Hospital, Zewditu Memorial Hospital and Menilik Referral Hospital.

The number of admitted neonates varies from time to time. The average annual admission rate was ranged from 1400 admission in Menilik Referral Hospital to 3,200 admissions in Gandhi Memorial Hospital (maternal and child health Hospital). The NICU had 48-bed capacity, 45-bed capacity, 40- bed capacity, and 34-bed capacity in Gandhi Memorial Hospital, Yekatit 12 Medical College Hospital, Zewditu Memorial Hospital and Menilik Referral Hospital respectively. In all selected Hospitals, NICU team receive babies who came with a complaint and assess if he/she fulfill admission criteria to the unit or not. In all units, there were continuous positive airway pressure machine, incubators, oxygen cylinders or oxygen concentrators, radiant warmers and Phototherapy machines.

### Study design and participants

Institutional prospective cohort study was undertaken between June 7^th^ and July 13^th^ 2021 in the NICU of the Addis Ababa public Hospitals. The enrollment of the participant was begun on June 7^th^, ended on July 7^th^ and the follow up of latest participant was closed on July 13^th^, 2021. Each enrolled neonate was followed until his/her age become seven days or until he/she develop event of interest (i.e., either death or censor).

#### Population

*Source population*. All neonates whose age were less than seven days and admitted to the NICU of Addis Ababa public Hospitals.

*Study population*. All neonates who were under the age of less than seven days old and admitted to NICU of selected Public Hospitals in Addis Ababa during study period.

#### Inclusion and exclusion criteria

*Inclusion criteria*. All less than seven days old neonates who were admitted to the corresponding neonatal intensive care unit of each selected Hospital.

*Exclusion criteria*. Neonate whose mother cannot communicate because of serious illness, mothers with mental problems, and neonates admitted without mothers.

### Sample size and sampling technique

Although all neonates consecutively admitted to the NICU of selected hospital during the study period were included in the study, the sample size was calculated for objective one and predictors to identify minimum required sample size. The minimum required sample size for a proportion of neonatal death was calculated using a single population proportion formula by considering the statistical assumptions of P = proportion of early neonatal mortality (21%), which was obtained from a study conducted in Amhara region [11], Z α/2 = corresponding Z score of 95% CI and d = margin of error (5%).

Therefore, $n=\frac{({1.96)}^{2}\times 0.21\times 0.79}{{\left(0.05\right)}^{2}}$ = 255. After the assumption of a 10% loss to follow-up, the final and minimum sample size required for the first objective was 281 neonates.

By considering the assumptions of significance level as (1-alpha) 95%, Power (1-beta) 80, Ratio of unexposed to exposed in sample 1, the adequacy of sample size for predictors is calculated by using Open-Epi software. The sample size was determined by using double population proportion formula by considering the death of Multiple Types of pregnancy, maternal education, Meconium aspiration syndrome, and Temperature of the neonate at admission as the major predictors in the previous studies [8, 11, 19] (Table 1). Therefore, the final sample size was that of objective two which was **332**.

**Table 1:** Sample size calculation summary for factors predicting early neonatal mortality.

Factors	P	HR	Sample size with 10% LTF	References
Multiple pregnancy	15	3.96	115	[11]
Temperature at admission(°c)	71.4	2.68	260	[8]
Maternal education	26	2.1	312	[11]
Meconium aspiration syndrome	17	2.24	332	[19]

P=Percent of Unexposed with Outcome.

**Fig 1:**
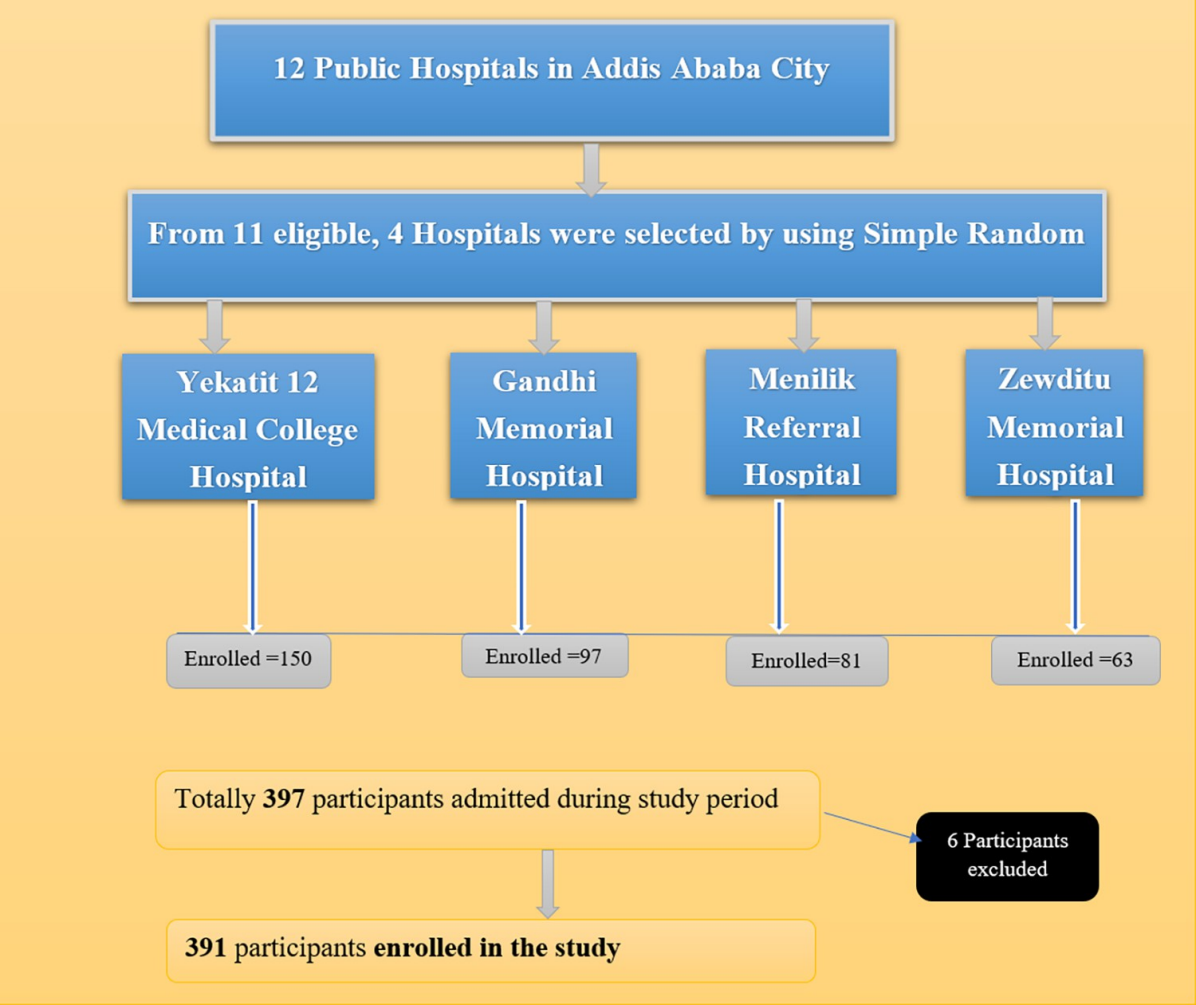
Sampling procedures of study conducted on time to death among neonates admitted to NICU in Addis Ababa public Hospitals, Ethiopia.

Four hospitals were randomly selected from available hospitals. All neonates consecutively admitted in selected hospitals’ NICUs were included in the study. The samples recruited this way can, with care, be reasonably representative ensuring that quantity of interest has no temporal or seasonal trends. It also reduce sampling bias for facility based study assuming that all admitted cases come randomly [20]. The minimum required sample size for each selected Hospital was allocated proportionally based on the number of its estimated previous case flows. The average of three months admission prior to enrollment was taken and minimum requirement was estimated. Accordingly, 128, 84, 71 and 50 minimum cases requirement were allocated for Gahandi memorial, Yekatit 12, Zewditu Memorial and Menilik II Referral Hospital respectively. Later on, the number of cases enrolled were 150, 97, 81 and 63 from Gahandi memorial, Yekatit 12, Zewditu Memorial and Menilik II Referral Hospital respectively (Fig 1).

### Data collection and quality assurance

Structured and an interviewer-administered questionnaires was used to collect the data. The questionnaire was adapted from the previous study [21]. During the data collection period, all early neonates fulfilling the inclusion criteria and admitted in NICU with their mothers were enrolled in the first seven days observation and then they were followed until the occurrence of the outcome of interest. Pre-recorded, patient-centered data were also collected through chart reviews or medical record review during enrolment of the participants and at the end of the study.

After putting aside the baseline information on socio-demographic factors, factors related to maternal, health service and care-related factors and pre-available neonatal factors on the first day of enrollment, gathering data on neonatal outcome was carried out every day throughout the follow-up period (i.e. from the time of admission to until the 7 days of neonatal age) or till the neonates will either develop an event of interest (i.e. death) or censored. For neonates who were discharged before the end of follow up time, the data collectors contacted the mother daily via a telephone call and inquire about the neonate’s condition and survival status.

In order to maintain the quality of the information, the questionnaire was developed in English and then it was translated to Amharic language by experts of the subject area and then it was translated back to English language. A Pretest was conducted on the 5% of the entire sample sizes in Tirunesh Beijing Hospital found in Addis Ababa before the beginning of actual data collection. Two nurses and two midwives who were not employed by the hospitals where the study participants were admitted served as the data collectors. Moreover, there were additional two supervisors. Training was given to the data collectors and supervisors regarding the ethical issues, general approaches, and strategies to minimize measurement bias.

Furthermore, each data collector had checked the questionnaires for completeness before leaving each study participant and each questionnaire was reviewed daily by supervisors and the principal investigator to check for completeness and clarity. Data entry format template was developed using Epidata 3.1 version. Data were entered and cleaned up before starting analysis.

### Variables

The dependent variable for this study was death of the neonate which was categorized as neonatal death (event of interest), and censored. The independent variables included: Socio demographic factors: age of the mother, women educational level, paternal educational level, residence, family members/birth interval, age of neonate, sex of the neonate, family income, gestational age, occupation. neonatal related factors: perinatal asphyxia, respiratory distress, sepsis, hypothermia, congenital anomalies, preterm birth, low birth weight (<2500g), low Apgar score, meconium aspiration syndrome, jaundice. gynecologic/obstetric and health services related factors: early initiation of breast feeding, antenatal care (ANC) visit, multiple pregnancies, skilled birth attendant, intervention (neonatal resuscitation and Kangaroo Mother Care), premature rupture of membrane, place of delivery, mode of delivery, parity, gravidity, hypertension during Pregnancy, HIV/AIDS and other medical disorders that are not related to pregnancy such as diabetic mellitus, heart disease etc.

### Statistical analysis

Data were coded, cleaned, edited, and entered into Epi data version 3.1 and then exported to STATA 14.0 version software for analysis. Descriptive statistics was employed. Texts, tables and figures had been also used to present data. Necessary assumptions of the Cox-proportional hazard regression model were checked both graphically and hypothetically using a hypothesis test called the Schoenfeld residual test (global test). Both bi-variable and multivariable Cox proportional hazard regression analyses were computed. Moreover, The Kaplan Meier survival curve was used to estimate survival time, and log rank test was used to compare the survival curves.

All variables having P-value ≤0.2 in the bi-variable analysis model were then further fitted to the multivariable model to identify independent predictors of early neonatal mortality and then the variables which have independent association with an outcome variable was identified based on AHR, with 95% CI and p-value ≤0.05. Accordingly, in the multivariable analysis, variables having P-value < 0.05 were considered as significant predictors of early neonatal mortality.

The overall global test of the full cox model was checked for proportional hazard assumption and it was met (p-value = 0.345). All covariates are met the proportional-hazard assumption. The goodness of fit of the model was also performed using the Cox-Snell residual test and showed that the model was adequate: the hazard function follows the 45° closed to the baseline. Additionally, Variance inflation factor (VIF) for all factors in the model was checked and it was less than 2.4. This suggested that the existence of multico-linearity problem in the model was less likely.

### Operational definitions

***Early neonatal death*:** is death of a neonate during the first seven days of age after birth (0 to <7days) [5].

***Event*: -** Death of a neonate within the 7 days after birth as evidenced by certified health profession confirmation or telephone verification from mothers.

***Censored*: -** Censored is the study subjects (neonate) who were not experience death during the follow-up period. It included neonates who were referred to other health institutions, lost to follow-up, discharged with improvement, or stayed with admission beyond 7 days of neonatal age.

***Time*: -** The period from starting of observation until the occurrence of death, lost to follow up, transferred out to other facilities, discharged within 7 days or end of the study measured in days.

**Other medical disorders**: Any maternal disease that is not related to pregnancy. It includes chronic disease like diabetic mellitus, heart disease and the like.

### Ethical clearance

All the methods and procedures carried out in this longitudinal study were in accordance with guidelines and regulation of Declaration of Helsinki and Hawassa University Institutional Review Board (IRB). Hawassa University College of Medicine and Health Science IRB approved the study with reference number of IRB 150/13. Besides, official permission was granted from Addis Ababa Health Bureau and each selected Hospital administration office. Because some mothers and guardians in Ethiopia could not read and write, we obtained verbal informed consent. The study’s purpose was explained using a standard information sheet. The participants were made aware that taking part in the study was completely voluntary and that they could refuse or withdraw from the study at any time. The study participants were assured that their refusal to participate in the study would not affect their right to use health services. Using an information sheet written in their language, the study’s details were read to the study participants. Participants’ names weren’t listed; instead, they were identified by their registration number. Additionally, the confidentiality and anonymity of data maintained. Verbal informed consent was recorded using a checkmark in information sheet.

## Results

### Characteristics of mothers and neonates

During follow up period, totally 397 neonates were admitted to neonatal intensive care unit. However, six (1.5%) neonates were excluded [two (0.5%) Orphaned and four (1%) incomplete information]. A total of 391 neonates with their mothers were involved in this study. The response rate was, therefore, 98.5%. These neonates were followed for minimum of two hours and for maximum of seven days. More than half, 229 (58.6%) of the neonates were male. Two-third, 269 (68.8%) of the neonates were admitted at age less than one day. Approximately two fifth of the neonates, 150 (38.4%), had birth weight less than 2500grams and 51 (13.1%) of the neonates have less than 1500grams at admission time (Table 2).

**Table 2:** Socio-demographic characteristics of neonate and neonates’ mothers admitted to neonatal intensive care unit of public Hospitals in Addis Ababa, Ethiopia, 2021 [n = 391].

Variable	Frequency	Percent	NDO	Death	IDR per 1000 NDO
**Sex of neonate**					
Male	229	58.6	1182	40	34
Female	162	41.4	862	28	33
**Age of neonate on admission**					
Less than 24 hours (one day)	79	20.2	261	8	31
Greater than or equal to one day	312	79.8	1783	60	34
**Weight of the neonate at admission**					
Greater than or equal to 4000	12	3.1	72	0	0
Normal (2500-3999)	229	58.6	1255	18	14
Low birth weight (1500-2499)	99	25.3	556	14	25
Very low birth weight (1000-1499)	41	10.5	145	27	186
Extreme low birth weight (< 1000)	10	2.6	15	9	600
**Age of the mother**					
less than 20	8	2.0	50	0	0
20-24	103	26.3	501	32	64
25-29	165	42.2	884	23	26
30-34	70	17.9	362	10	28
35-39	39	10.0	211	3	14
40 or more	6	1.5	35	0	0
**Marital status of the mother**					
Single	14	3.6	74	2	27
Married	364	93.1	1915	64	33
Divorced	10	2.6	44	2	45
Widowed	3	0.8	12	0	0
**Occupation of the mother**					
House wife	137	35.0	728	23	32
Self/private employee	148	37.9	739	33	45
Merchant	54	13.8	66	0	0
Government employee	31	7.9	316	6	19
Others	21	5.4	196	3	15
**Educational status of the mother**					
Unable to read and write	20	5.12	111	2	18
Read and write	22	5.63	103	6	58
Grade 1-8	165	42.2	862	34	39
Grade 9-12	129	32.99	647	25	39
College and above	55	14.07	322	1	3
**Educational status of the father**					
Unable to read and write	14	3.6	77	1	13
Read and write	14	3.6	68	3	44
Grade 1-8	94	24.0	472	21	45
Grade 9-12	148	37.9	739	38	51
College and above	121	30.9	689	5	7
**Place of residence**					
Urban	372	95.14	1952	65	33
Rural	19	4.86	93	3	32
**Family size**					
Two or less	138	35.3	705	34	48
Three or more	253	64.7	1340	34	25
**Monthly income**					
≤3000	106	27.1	536	23	43
3001-8000	190	48.6	1024	27	26
>8000	95	24.3	485	18	37

**Keys:** NDO=neonatal-day observed, IDR=Incidence density rate

Regarding socio-demographic status of the mother, about 235(70%) of the mothers were found in age group between 20 and 29 years. More than nine in ten of neonates’ mother, 364(93.1%), were married. Besides, nearly two third, 250 (64%), of the mothers were followers of Orthodox Christian. Concerning occupational status of the mother 137 (35.0%) of them were housewives and 148 (37.9%) of them were self/private employee (Table 2).

About half, 184 (47%), of the mother and 269 (68.8%) of the father were attended education until high school or more. Nearly two out of three, 253 (64.7%), of the respondents have three or more family size. Concerning place of residence, 372 (95%) of the respondent were urban residents (Table 2).

### Maternal obstetric and health services related characteristics

In this study, half of the mothers, 195 (49.9%), were multipara. Regarding mothers who had previous delivery history (n=209), about half, 110 (53%), had two to four birth intervals. Furthermore, majority 338 (86%) of the mother had three or more ANC visit during recent pregnancy while 13 (3.30%) of the mothers had no history of ANC visit. Almost all of the neonates, 374 (98.2%), were born at health facility among who 257 (65.7%) were born at Hospital. Regarding mode of delivery, more than half, 213 (54.5%), of the mothers were given birth through spontaneous vertex delivery (SVD) and 153 (39.1%) of the mothers were given birth through cesarean section or by operation. Among the delivered neonates, 356 (91.0%) were born single type of birth (Table 3).

**Table 3:** Maternal and health services related characteristics of mothers of neonates admitted to neonatal intensive care unit of public Hospitals in Addis Ababa, Ethiopia, 2021 [n = 391].

Variable	Frequency	Percent	NDO	Death	IDR per 1000 NDOs
**Number of ANC visit**					
Four times or more visit	238	60.87	1289	20	16
Three times visit	100	25.58	526	24	46
Two times visit	36	9.21	138	20	145
One time visit	4	1.02	17	2	118
No visit	13	3.32	75	2	27
**History of Previous pregnancy**					
Yes	57	14.58	256	23	90
No	234	85.42	1789	45	25
**Parity**					
Prim-para	182	46.5	944	38	40
Multipara	195	49.9	1018	29	28
Grand multipara	14	3.6	83	1	12
**Birth interval b/n this birth and before (n=234)**					
Less than 2 years	11	4.7	33	6	181
2-4 years	158	67.5	827	26	31
More than four years	65	27.8	354	4	11
**Place this neonate delivered**					
Health center	127	32.5	620	24	39
Hospital	257	65.7	1394	42	30
Home or on road	7	1.8	31	2	65
**Types of birth**					
Single	356	91.0	1856	56	30
Multiple	35	9.0	189	12	63
**Mode of delivery**					
SVD (Spontaneous vertex delivery)	213	54.5	1064	41	39
Instrumental delivery	25	6.4	141	1	7
Cesarean section	153	39.1	840	26	31
**Hypertension during Pregnancy**					
Yes	57	14.6	255	23	90
No	334	85.4	1790	45	25
**Bleeding during pregnancy**					
Yes	28	7.2	135	7	52
No	363	92.8	1910	61	32
**HIV status**					
Positive	29	7.4	122	10	82
Negative	362	92.6	1923	58	30
**Diabetic mother**					
Yes	10	2.6	52	1	19
No	381	97.4	1993	67	34
**Anemia during current pregnancy**					
Yes	95	24.3	474	16	33
No	296	75.7	1571	52	33
**Premature rupture of membrane**					
Yes	36	9.2	212	5	24
No	355	90.8	1833	63	34
**Abortion history**					
One abortion	75	19.2	376	15	40
Two or more history of abortion	12	3.1	64	1	16
No history of abortion	304	77.7	1605	52	32
**Previous history of neonatal death?**					
Yes	24	6.1	139	2	14
No	367	93.9	1906	66	35
**Place of current neonate birth**					
Inborn (in study site)	233	59.6	1286	40	31
Out born (out of this site)	158	40.4	759	28	37

**Keys:** NDO=neonatal-day observed, IDR=Incidence density rate

The common obstetrical complications encountered during current pregnancy were: presence of anaemia during current pregnancy 95 (24.3%), pregnancy-induced hypertension 57 (14.6%), premature rupture of membrane 36(9.2%), HIV infection 29 (7.4%), ante partum haemorrhage 28 (7.2%), diagnosed STI/ UTI 28 (7.2%), and history of neonatal death 24 (6.1). Another finding is that 233 (60%) of the neonates were born at hospitals where the study was conducted done (Table 3).

### Neonatal characteristics

Among the neonates admitted to neonatal units and involved in the study, three forth 296(75.7%) of them had cried immediately at birth. Regarding Apgar score; 158 (40.4%) of the neonates had low first minute Apgar score while 80 (20.5) of them had low fifth minute Apgar score. Moreover, half of them, 195 (49.9%), had normal temperature while still near to half of them, 179 (46%), had moderate hypothermia at admission to NICU. One hundred forty-one (36.1%) of the neonates were resuscitated at birth. Regarding breast feeding initiation and continuation about one fourth 107 (27.4%) of the neonate initiated exclusive breast feeding (EBF) within the first one hour of birth; however, 180 (46%) were ever not started EBF during data collection (Table 4).

**Table 4:** Neonatal characteristics of neonates admitted to neonatal intensive care unit of public Hospitals in Addis Ababa, Ethiopia, 2021 [n = 391].

Variable	Frequency	Percent	NDO	Death	IDR per 1000 NDOs
**Did the newborn cry immediately at birth?**					
Yes	296	75.7	1624	25	15
No	95	24.3	421	43	102
**Score of the first minute Apgar score**					
Low (<7)	158	40.4	777	54	69
Normal (≥7)	233	59.6	1268	14	11
**Score of the 5thminute Apgar score**					
Low (less than 7)	80	20.5	315	43	137
Normal (≥7)	311	79.5	1729	25	14
**Temperature of the neonate at admission (** ^**o**^ **C)**					
Hyperthermia	14	3.58	60	1	16
Normal	195	49.87	1056	18	17
Mild and moderate hypothermia	179	45.78	912	48	5
Severe hypothermia	3	0.77	17	1	59
**Did the newborn resuscitate at birth?**					
Yes	141	36.1	657	52	79
No	250	63.9	1388	16	12
**Did the newborn being kept under KMC**					
Yes	25	6.4	138	7	51
No	366	93.6	1907	61	32
**Did the Neonate initiate EBF?**					
Yes	218	54.0	1174	14	12
No	180	46.0	870	54	62
**When did the neonate start EBF**					
within one hour	107	27.4	567	3	5
After one hour	104	26.6	596	3	5
Not ever initiated	180	46.0	882	62	70
**Feeding of the newborn during data collection days**					
Only breast milk	211	54.0	1162	7	6
Breast milk with additional fluids ^***a***^	40	10.2	229	5	22
Maintenance fluid	129	33.0	592	55	93
Formula feeding	11	2.8	62	1	16
**Newborn have a sepsis**					
Yes	194	49.6	1007	33	33
No	197	50.4	1038	35	34
**Newborn have respiratory distress syndrome**					
Yes	172	44.0	911	44	48
No	219	56.0	1134	24	21
**Newborn have asphyxia**					
Yes	70	17.9	367	21	57
No	321	82.1	1678	47	28
**Newborn have jaundice**					
Yes	77	19.7	398	2	5
No	314	80.3	1707	66	39
**Other neonatal complications (n=99)**					
Meconium aspiration syndrome	32	8.2	199	3	15
Congenital anomaly	30	7.7	171	1	6
ABO incompatibility or setup	19	4.9	113	2	18
Hypoglycemia	10	2.6	50	3	60
Birth trauma	8	2.0	40	1	25
**Gestational age of current neonate**					
Very preterm	60	15.3	232	37	159
Moderate to late preterm	56	14.3	297	13	44
Term	269	68.8	1489	18	12
Unknown	6	1.5	27	0	0

**Keys:** NDO=neonatal-day observed, IDR=Incidence density rate, KMC= Kangaroo Mother Care

^***a***^ Additional fluids include any fluids other than formula feeding and maintenance fluid. It includes fluid for treatment.

Different neonatal complications were diagnosed during admission to the neonatal intensive care unit. The common neonatal complications diagnosed at admission were: early neonatal sepsis 194 (49.6%), respiratory distress syndrome (RDS) 172 (44.0%), perinatal asphyxia 70 (17.9%), neonatal jaundice 77 (19.7%), congenital anomaly 30 (7.7%), Meconium aspiration syndrome 32 (8.2%). One hundred and sixteen (29.6%) of the neonates were born before 37 weeks of gestational age (born preterm). From these preterm neonates, 60 (15.5%) of them were born at very preterm (less than 32 weeks) gestational age (Table 4).

### Incidence of the early neonatal mortality

A total of 391 of neonates were followed for a minimum of two hours and a maximum of seven days, with a median (IQR) follow up time of 6.22 (4-6.91) days. During the follow up period, 68 (17.4%) of them were died. The total time at risk for 391 neonates was 2045 person-days with an incidence rate among admitted neonate 33.25 per 1000 neonate days observation (95% CI: 26.22,42.17). In addition, 12 (3.1%) were lost from the follow up, 5(1.3%) were transferred out to other hospitals due to surgical and shortage of bed, and the other 306 (78.3%) were alive.

**Fig 2:**
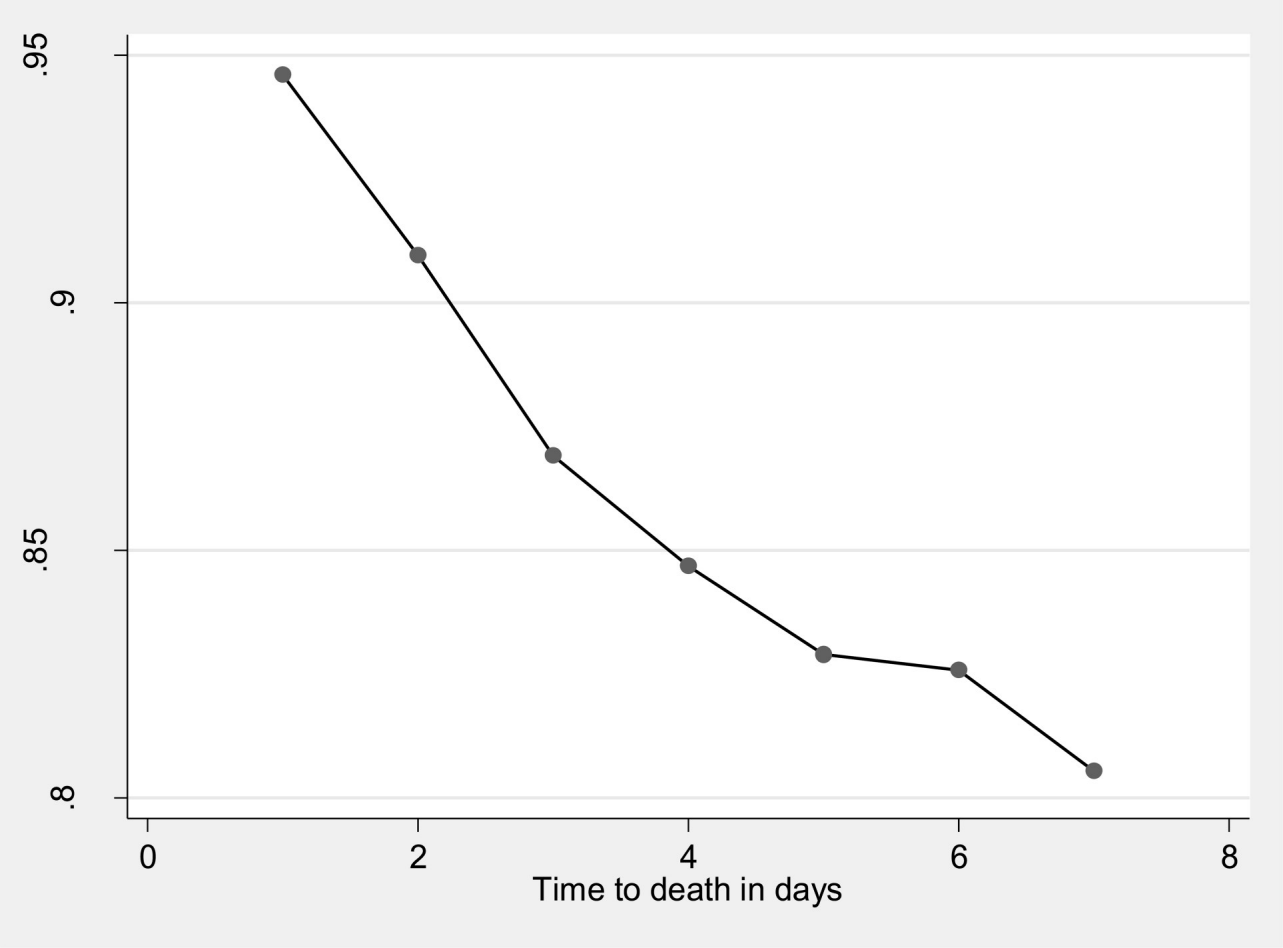
Kaplan Meir survival estimate of cumulative Survival status of the early neonates during follow up time in the Addis Ababa public Hospitals, central Ethiopia, 2021.

**Fig 3:**
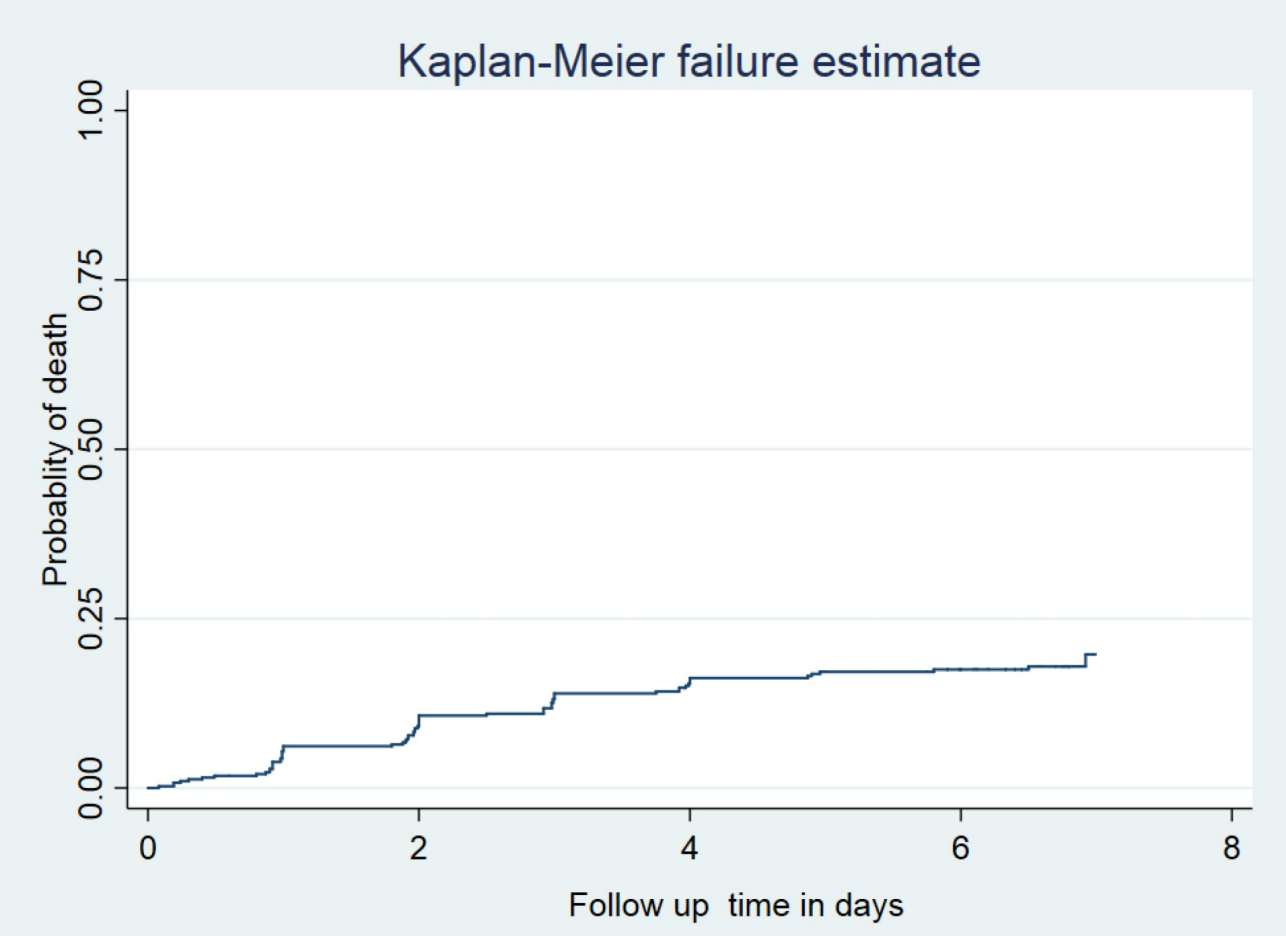
Kaplan Meir cumulative failure function of the early neonates during follow up time in the Addis Ababa public Hospitals, central Ethiopia, 2021.

Our data also showed that 8 (11.8%) of the neonates were died within the first 24 hours of follow up; 47 (69.1%) were died between first and third days and, 13 (19%) of them died after three days of follow up. This result highlights that 55 (81%) of the dead neonates were died within the first seventy-two hours (three days) of follow up. The cumulative proportion of survival at the end of the first, second, third, fourth, fifth, and sixth days was 94.6%, 91.0%, 87%, 85%,83%, and 82.6% respectively (Figs 2 and 3).

**Fig 4:**
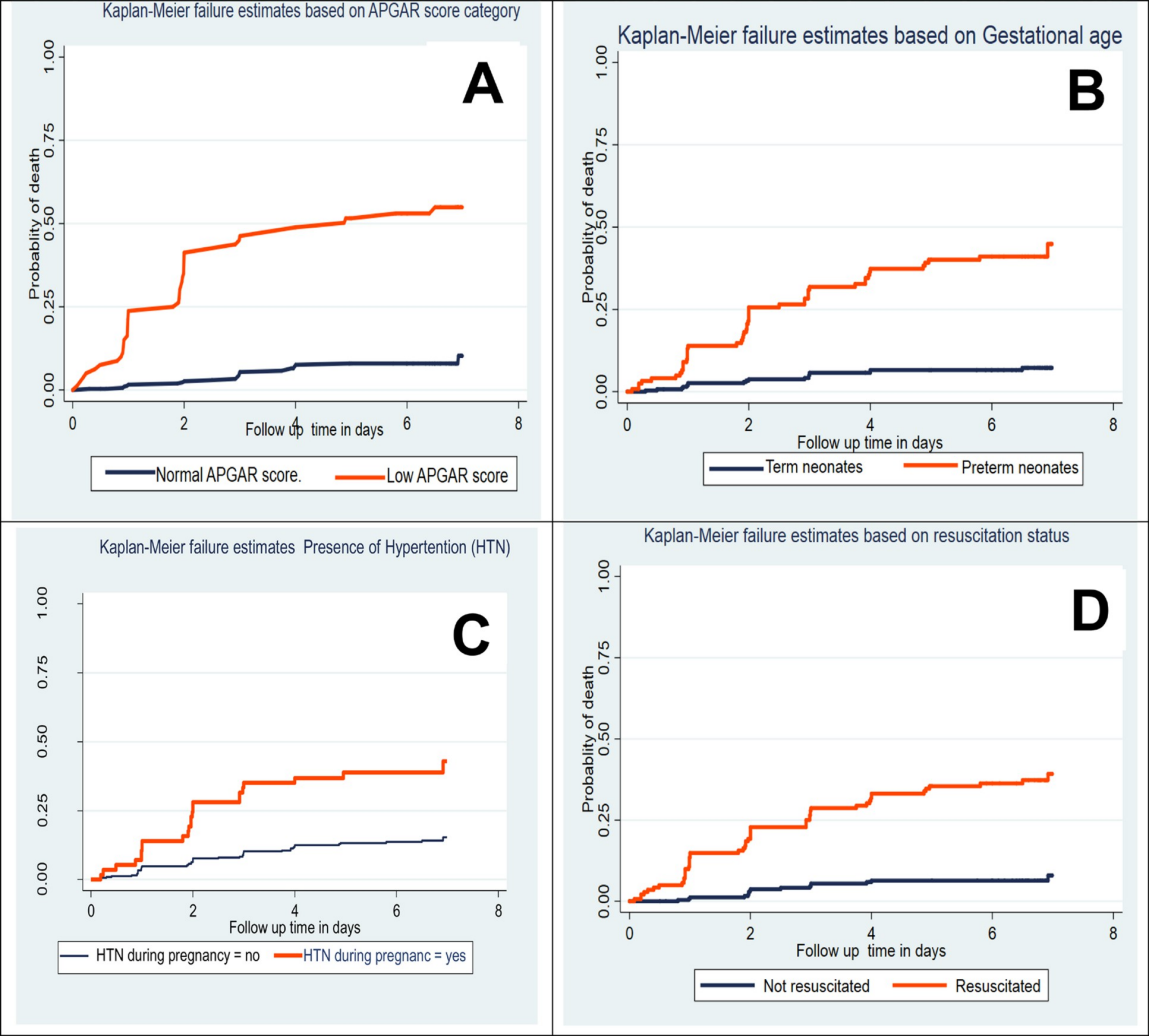
Kaplan-Meier failure estimate of early neonates based on Apgar PGAR score (A), gestational age (B), presence of hypertension during pregnancy (C) and resuscitation status (D) among neonate admitted to neonatal intensive care unit in Addis Ababa public hospitals Ethiopia, 2021.

Kaplan–Meier survival curve together with the log-rank test was fitted to test for the presence of a difference in the occurrence of death among the categorical explanatory variables. Accordingly, the incidence of mortality was higher among preterm neonates compared with term neonates, neonates whose mother had pregnancy induced hypertension have higher mortality than normotensive mother, neonate with low five-minute Apgar score have higher death compared with normal fifth minute Apgar score neonate, and neonate who did not initiate exclusive breast feeding was higher mortality compared to their counterpart (Fig 4, Table 5).

**Table 5:** Comparisons of death among different levels of predictor variables using the log-rank test for neonate admitted to NICU of Addis Ababa public Hospitals, Ethiopia; 2021.

Variable	Log-rank (χ2)	p-Value
Gestational age	70.23	<0.001
Hypertension during Pregnancy	25.69	<0.001
5^th^ min. Apgar score	49.70	<0.001
Initiate EBF	62.79	<0.001
cry at birth	71.04	<0.001
1^st^ min. Apgar	109.74	<0.001
Birth interval	18.28	<0.001
Mother HIV status	7.72	0.0055
Type of current birth	6.23	0.013
T° of the neonate	13.91	0.002
Admission weight	41.38	<0.001
Neonate has RDS	12.28	0.005
Age of the mother	13.87	0.0010

### Predictors of early neonatal mortality

In the bi-variable analysis of selected variables: family size, gestational age, ANC visit, Hypertension during Pregnancy mother HIV status, type of current birth, temperature of the neonate, first minute Apgar score, five-minute Apgar score, birth weight at admission, cry immediately at birth, Initiation of exclusive breast feeding, neonatal RDS, presence of birth asphyxia, parity and Resuscitation were shown association with time to death at a p-value < 0.2. However, in the final multivariable Cox-proportional hazard model; gestational age, resuscitation, five-minute Apgar score, temperature of the neonate and crying immediately at birth were found to be independent predictors of neonatal death (Table 6).

**Table 6:** Predictors of early neonatal death among neonates admitted to NICU in Addis Ababa public Hospitals, Ethiopia 2021 (n =391).

Variables	Survival status		CHR (95%CI)	P-value	AHR (95%CI)	P-value
	Death #(%)	Censored # (%)				
**Family size**						
≤2	34 (24.6)	104 (75.4)	1.86 (1.15,3.0)	0.011	1.02 (0.5,2.1)	0.521
≥3	34 (13.4)	219 (86.6)	1		1	
**N****o** **of ANC visit**						
≥3	44 (13)	294 (87)	0.25 (0.15,0.41)	<0.001	0.8 (0.46,1.5)	0.650
≤2	24 (45.3)	29 (54.7)	1		1	
**Gestational age**						
<37week	50 (41)	72 (59)	7.02 (4.1,12.04)	<0.001	6.0 (2.02,17.50)	<0.001
≥37week	18 (6.7)	251 (93.3)	1		1	
**Hypertension during Pregnancy**						
Yes	23 (40.4)	34 (59.6)	3.34 (2.02,5.6)	<0.001	1.63 (0.90, 2.94)	0.221
No	45(13.5)	289 (86.5)	1		1	
**HIV status**						
Positive	10 (34.5)	19 (65.5)	2.47 (1.30,5.00)	0.006	1.82(0.82,4.10)	0.263
Negative	58 (16)	304 (84)	1		1	
**Temperature of the neonate**						
< 36.5	49 (26.9)	133 (73.1)	3.13 (1.8,5.19)	<0.001	2.67 (1.41,5.02)	0.007
≥36.5	19 (9.1)	190 (90.9)	1		1	
**1**^**st**^ **min. A**pgar **score**						
<7	54 (34.2)	104 (65.8)	6.40 (3.53,11.45)	<0.001	1.10 (0.48,2.57)	0.342
≥7	14 (6)	219 (94)	1		1	
**Resuscitation**						
Yes	52 (36.9)	89 (63.1)	6.60 (3.77,11.56)	0.010	2.80 (1.51,5.10)	<0.001
No	16 (6.4)	234 (93.6)	1		1	
**5**^**th**^ **min. A**pgar **score**						
<7	43 (53.8)	37 (46.3)	9.10 (5.54,14.9)	<0.001	3.93 (1.5,6.77)	<0.001
≥7	25 (8.0)	286 (92.0)	1		1	
**Birth weight**						
≥2500	18 (7.5)	223 (92.5)	1		1	
<2500	50 (33.3)	100 (66.7)	4.94 (2.88,8.48)	<0.001	1.16 (0.4,3.35)	0.820
**Cry at birth**						
Yes	25 (8.4)	271 (91.6)	0.16 (0.09,0.26)	<0.001	0.48 (0.26,0.87)	0.013
No	43 (45.3)	52 (54.7)	1		1	
**Initiate EBF**						
No	54 (31.2)	119 (68.8)	5.30 (2.94, 9.53)	0.005	1.80 (0.94,3.44)	0.181
Yes	14 (6.4)	204 (93.6)	1		1	
**Maternal age**						
<25	32 (28.8)	79 (71.2)	1		1	
25-29	23 (13.9)	142 (86.1)	0.46 (0.27,0.79)	0.004	0.54 (0.28, 1.04)	0.123
≥30	13 (11.3)	102 (88.7)	0.37 (0.2,0.71)	0.002	0.60 (0.27,1.32)	0.91
**Has RDS**						
Yes	44 (25.6)	128 (74.4)	2.36 (1.44,3.88)	0.015	1.16 (0.6,1.75)	0.650
No	24 (11)	195 (89)	1		1	
**Birth asphyxia**						
Yes	21 (30)	49 (70)	2.09 (1.25,3.50)	0.051	1.1 (0.56,2.15)	0.550
No	47 (14.6)	274 (85.4)	1		1	
**Parity**						
Primipara	38 (20.9)	144 (79.1)	1.47 (0.91,2.37)	0.114	1.33 (0.711, 2.07)	0.450
Multipara	30 (14.4)	179 (85.6)	1		1	

ANC=Antenatal Care, HIV=Human Immunodeficiency Virus, Apgar =Appearance, Pulse, Grimace, Activity, and Respiration, EBF= Exclusive Breast Feeding, RDS= respiratory distrust syndrome

The hazard of death among preterm (premature) neonates were six times [AHR: 6 (with 95% CI 2.02, 17.50)] higher than term neonates. The hazard of death among neonates with low fifth minute Apgar score was 3.93 times [AHR: 3.93 (95% CI; 1.50, 6.771)] higher than those with normal Apgar score. The hazard of neonatal mortality among resuscitated neonates were 2.80 times [AHR: 2.8 (95% CI 1.51, 5.10)] higher than not resuscitated neonates. The hazard of death among neonates with hypothermia were 2.67 times [AHR: 2.67 (95%CI; 1.41, 5.02)] higher than those with normal temperature neonates. The hazard of death among neonate who cried immediately at birth were 52% [AHR: 0.48 (95%CI; 0.26, 0.87)] lower than those who do not cried (Table 6).

#### Assessment of model adequacy

**Fig 5:**
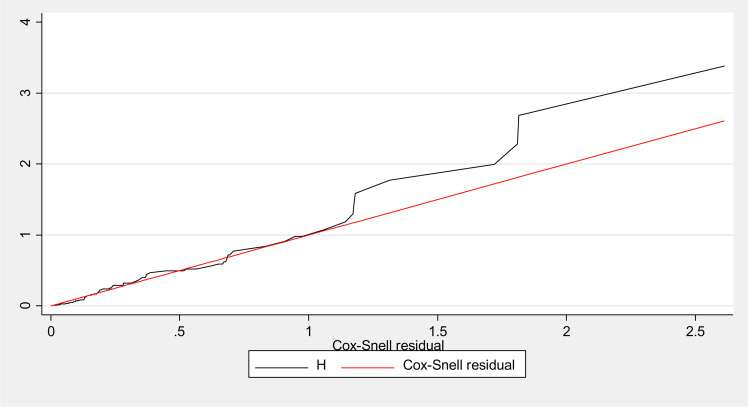
Assessment of goodness of fit of the model by Cox-Snell residual Nelson-Alen cumulative hazard graph.

The overall global test of the full cox model was checked for proportional hazard assumption and it was met (p-value = 0.345). All covariates met the proportional-hazard assumption. The goodness of fit of the model was also performed using the Cox-Snell residual test and showed that the model was adequate: the hazard function follows the 45° closed to the baseline (Fig 5).

## Discussion

This study aimed to assess time to death of early neonate and its predictors of mortality among neonates admitted to neonatal intensive care unit of Addis Ababa public Hospitals. Our results demonstrated that the incidence density among admitted neonate in this study was 33.25 per 1000 neonate day’s observation (95% CI 26.22, 42.17). This is higher than study conducted in Mekelle general and Ayder comprehensive specialized Hospital (22.45 death per 1000 neonates-days observation) [22].The reason for the variation could be due to sample size. Besides, the quality of health care services and facilities may be the cause of the variation. Quality of service at hospitals may vary and determine the magnitude of neonatal mortality [23]. Therefore, it is possible that some hospitals in Addis Ababa may have lower quality of service or resources than those in Mekelle and Ayder, which could affect the survival of neonates.

However, this finding is alike with study conducted in Amhara regional state referral hospitals [11] and Debre Markos referral Hospital, in which 39.6 early neonatal death per 1000 neonate-days was observed [24].

Moreover, higher incidence rate was reported in Wolayita Sodo (77 neonatal deaths per 1000 neonate-days) than this finding [19]. Possible justification for this variation could be due to variation in sources of the data. The study conducted in Wolayita Sodo was secondary data (retrospective cohort). The nature of the study design pose for inaccurate information, and systematic bias may have been introduced. Furthermore, the level of income, comforts and services available, which generally applied to a society, may also differ in two areas.

Low Apgar score in the 5^th^ minute after birth was found to increase the likelihood of neonatal mortality in this study. This finding is affirmed by previous findings which depicted low Apgar scores have a higher predictive value of neonatal morality [6, 8, 24]. The justification for this relation is that low Apgar score is resulted from extrauterine complications. If the baby has a low Apgar score, which is defined as 7 or below, it may has signs such as a slow heart rate or no heart rate, weak breathing or no breathing, little flexion or no muscle tone, little to no response to stimulation, and little to no color, which means poor blood flow or circulation [25].

The hazard of early neonatal mortality was higher among hypothermic neonates as compared with those who have normal temperature. This finding is consistent with other studies conducted at Jigjiga University Referral Hospital, Eastern Ethiopia [26], University of Gondar comprehensive specialized hospital, Northwest Ethiopia [27] and one global pooled evidence [28]. This may be due to environmental factors like delivery area, maternal complications like cesarean delivery. It could also be due to neonatal disorders like sepsis or a combination of environmental factors and neonatal disorders which pose neonate for bacterial infection, hypoglycemia and hypothermia.

Crying after delivery was another factor that associated with decreased hazard of early neonatal mortality. Cried neonates were less likely to die than those who were not cried. This result is in line with study conducted at University of Gondar comprehensive specialized hospital, Northwest Ethiopia [27] and Nekemte Referral Hospital, western Ethiopia [29]. The possible reason could be unable to crying has another underlining problem.

Unlike many of the factors that take Childrens’ lives, the risk to children from premature birth continues to affect all nations globally [4]. The same thing is found in this study which showed that the hazard of death of preterm babies was higher than neonates born on the due date. This finding is in agreement with other studies conducted in Sub-Saharan Africa, eastern Ethiopia, public hospitals of Gamo and Gofa zones, southern Ethiopia, Jimma, South West Ethiopia, Ayder Comprehensive Specialized Hospital, Northern Ethiopia and in India, Nepal and Bangladesh [6, 8, 21, 30–32]. This could be due to the fact that preterm newborns are at greater risk of death, which could be resulted from anatomical and physiologic immaturities. For babies born preterm (before 37 weeks) the risk is further multiplied by health complications due to underdevelopment of organs, muscles and immune systems. Therefore, they were more likely to be prone to complications such as hypothermia, infections, and birth asphyxia resulting in tissue hypoxia and multi-organ failure) [17].

Another factor that predicted early neonatal mortality in this study was resuscitation of the neonate. The hazard of death of early neonate who resuscitated was higher than those not resuscitated neonate. This finding is consistent with other study conducted in Southern part of Ethiopia [19], Teaching Hospitals of Addis Ababa University [33] and survey from all Ethiopian public hospitals, health centers and private health facilities [34]. The reason could be due to the fact that neonates who need resuscitation are those with complications and at high risk to death. Another reason could be neonatal resuscitation intervention may be ineffective. The Ethiopian public health institute report found that there is very low-quality evidence of effectiveness or lack of effectiveness of neonatal resuscitation intervention in Ethiopia [35].

What is more; unlike many studies conducted in different parts of Ethiopia and other sub-Saharan Africa, ANC visit [19, 21, 36, 37], multiple pregnancy [11, 19, 36, 37] and place of residence [6, 19, 36, 38] were not significantly predict neonatal death in this study area. This could be due to the fact that there is highest ANC coverage in Addis Ababa city. ANC coverage from a skilled provider may play a role in reducing neonatal death due to pregnancy complications like multiple births. The contrary may also due to sociodemographic variation between Addis Ababa and other sites [7, 9].

The fact that this study was conducted prospectively is its key strength. As a result, it was possible to include a variety of sociodemographic, obstetric, and neonatal characteristics that were critical in determining predictors of neonatal death. Furthermore, it was performed on all admitted neonates who met the inclusion criteria without sampling, removing the chance of sampling error. However, the study had certain drawbacks. The study’s limitation is that neonates who died before being admitted to the neonatal intensive care unit and those without a parent were not included. This could lead to an underestimation of neonatal mortality. Furthermore, the proficiency and training level of health workers, which could affect the neonate’s survival status, is not assessed.

## Conclusion

The incidence of early neonatal mortality in Addis Ababa public Hospitals was highFive-minute Apgar score, preterm birth, crying at birth, low temperature of the neonate during admission and being resuscitated were factors predicting early neonatal death in Addis Ababa public Hospitals.

To decrease the high burden of early neonatal mortality, designing strategies which can address all these preventable predictors is recommended. Promote engagement of and empower mothers, families and communities to participate in and demand quality care for neonates not crying, preterm, hypothermic and newborns with low fifth minute Apgar score. Since post-natal care is critical during the first hours after birth and important throughout the first week of life, immediate evaluation of the neonate at birth and attention to neonate with low Apgar score, not crying, low body temperature and premature. Furthermore, there should be emphasizing for neonatal resuscitation. We recommend prospective longitudinal researches to be done in the respective Addis Ababa city including those who are not admitted to Neonatal intensive care unit and at the community level to identify all possible problems that impede neonatal survival in Addis Ababa. Additionally, it is better if health care proficiency and training status is assessed.

## Supporting information

S1 File Questionnaire used to collect data. (DOCX)

S1 Data Dataset related to this study. (DTA)

## Abbreviations

ANC: Antenatal Care
NICU: Neonatal Intensive Care Unit
RDS: Respiratory Distress Syndrome
