# Supplementary material for: Time to death and predictors of mortality among early neonates admitted to neonatal intensive care unit of Addis Ababa public Hospitals, Ethiopia: Institutional-based prospective cohort study
Source: PLoS One. 2024 Jun 6;19(6):e0302665. doi: 10.1371/journal.pone.0302665 (PMC11156352; doi:10.1371/journal.pone.0302665)
Supplement: S1 File — (DOCX) [file pone.0302665.s001.docx]

**Annex I: English version questionnaire**

**Introduction:**

My name is ------------------. I am working as a data collector in a research conducted by Erean Shigign (MPH in Epidemiology student at Hawassa University). We are interviewing women who gave birth and their neonates are admitted to Neonatal Intensive care Unit in Addis Ababa public hospitals including this hospital. To attain this purpose, your honest and genuine participation by responding to the question prepared is very important & highly appreciated.

**Confidentiality and consent**

I would like you to answer some personal questions that some people may find difficult to answer. Your answers are completely confidential. Your name will not be written on this form. Other people will not be told what you said in connection to your name.

You do not have to answer any question if you don’t want to and you can stop the interview at any time. However, your honest answer to these questions will help us to better understand the experience of mothers related to early neonatal mortality. We would greatly appreciate your help in responding to this study. After baseline information from you, we will follow up on the status of your neonate for 7 days and if you are told to go home, we will call you daily until the so-called day is up!

Would you be willing to participate?

If yes, proceed

If no, thank and stop here.

Day and time the study started___________________.

**Part I: Socio demographic characteristics**

| S.No | Variables | Coding Categories | Skip |
| --- | --- | --- | --- |
| 101 | Age of Neonate at admission | ------------(in Days) |  |
| 102 | Sex of Neonate | 1. Male 2. Female |  |
| 103 | Weight of the neonate at admission | ___________ |  |
| 104 | Age of the Mother | __________ |  |
| 105 | Marital status | 1. Single 2. Married 3. Divorced 4. Widowed |  |
| 106 | Religious status | 1. Orthodox 2. Muslim 3. Protestant 4. Others |  |
| 107 | Educational status of the mother | 1. Unable to read and write 2. Read and write 3. Grade 1-8 4. Grade 9-12 5. College and above |  |
| 108 | Educational status of the father | 1. Unable to read and write 2. Read and write 3. Grade 1-8 4. Grade 9-12 5. College and above |  |
| 109 | Maternal occupational status | 1. House wife 2. Self-employee 3. Farmer 4. Merchant 5. Others (specify) |  |
| 110 | Family size | __________ (in number) |  |
| 111 | Monthly Income | __________ |  |
| 112 | Place of residence | 1. Urban 2. Rural |  |
| 113 | Maternal habit of alcohol intake | 1. Yes 2. No |  |
| 114 | Maternal habit of chewing khat | 1. Yes 2. No |  |

**PART-II; Maternal and health services related Factors**

| S.No | Variables | **Coding Categories** | Skip |
| --- | --- | --- | --- |
| 201 | Does the mother have ANC f/up for this birth? | 1. Yes 2. No | If 2 go to 203 |
| 202 | How many visits did she attend? | 1. One visit 2. Two visits 3. Three visits 4. Four visits |  |
| 203 | Does the mother have the previous history of pregnancy? | 1. Yes 2. No | If 2 go to207 |
| 204 | How many Total pregnancies does she have (Gravidity)? | ________________ |  |
| 205 | How many alive births does she have (Parity)? | ___________ |  |
| 206 | What was the birth interval b/n the current birth and birth before this birth? | ________ Years |  |
| 207 | Where did the mother deliver? | 1. Health institution 2. Home |  |
| 208 | Types of birth | 1. Single 2. Twin 3. other |  |
| 209 | Who did attend the birth? | 1. Relatives 2. TTBA 3. HEW 4. Health professionals |  |
| 210 | What was the mode of delivery? | 1. Spontaneous vaginal delivery 2. Assisted instrumental delivery 3. Cesarean section |  |
| 211 | Did the mother have pregnancy-induced hypertension? | - 1. Yes   2. No |  |
| 212 | Did the mother have bleeding during pregnancy? | - 1. Yes   2. No |  |
| 213 | Did the mother have a history of STI? | 1. Yes 2. No |  |
| 214 | Did the mother have a history of UTI | 1. Yes 2. no |  |
| 215 | Did the mother have an intrapartum fever? | 1. Yes 2. No |  |
| 216 | Did the mother have a history of abortion? | 1. Yes 2. No |  |
| 2017 | If yes for question 2016, what kind of abortion was it? | - 1. spontaneous abortion   2. induced abortion   3. any other (specify) |  |
| 218 | What was the gestational age of the current newborn? | ____________ (in weeks) |  |
| 219 | Mother HIV infection status? | 1. positive 2. negative |  |
| 220 | Did the mother have diabetes mellitus? | 1. Yes 2. No |  |
| 221 | What was the hemoglobin level of the mother? | ____________ |  |
| 222 | Did the mother have history of neonatal death previously? | 1. yes 2. 2. No |  |
| 223 | Any other maternal complications? Specify | ________ |  |
| 224 | Place of current birth | 1. Inborn (in study site)  2. Out born(out of this site)  3.from home |  |

**PART-III Neonatal Factors**

| **S.No** | **Variables** | **Categories** | Skip |
| --- | --- | --- | --- |
| 301 | Did the newborn cry immediately at birth? | 1. Yes 2. No |  |
| 302 | What is the score of the first minute APGAR score? | ____________ |  |
| 303 | What is the score of the 5^th^minute APGAR score? | ------------------- |  |
| 304 | The temperature of the neonate at admission in °C | ___ |  |
| 305 | Did the newborn resuscitate at birth? | 1. Yes 2. No |  |
| 306 | Does the newborn being kept under KMC within one hour? | 1. Yes 2. No |  |
| 307 | Does the Neonate initiate EBF? | 1. Yes 2. No | If 2 go to 309 |
| 308 | When did the neonate start EBF? | 1. Within 1 hour 2. After 1 hour |  |
| 309 | What was the feeding of the newborn within the first 7 days? | 1. Only breast milk 2. Withadditional food 3. Other(specify) |  |
| 310 | Does the newborn have a sepsis? | 1. Yes 2. No |  |
| 311 | Does the newborn have respiratory distress syndrome? | 1. Yes 2. No |  |
| 312 | Does the newborn have asphyxia? | 1. Yes 2. No |  |
| 313 | Does the newborn have jaundice? | 1. Yes 2. No |  |
| 314 | Does the newborn have congenital anomaly? | 1. Yes 2. No |  |
| 315 | If yes for question 314, specify the anomaly. |  |  |
| 316 | Any other neonatal complications? Specify | ___________ |  |

- - - Date of birth ____________/__________/____________
    - Date of Admission --------------/-----------------/-----------------
    - Date of study participant was Died -----------/----------/-----------------
    - Date of study participant lost to follow up -----------/----------/--------
    - Date of the study participant with draw the treatment_________/_______/________
    - Date of the study Participant transferred out_________/_________/__________
- Date the follow up finished______________
  - - Total No of days the neonates has been followed___________ Day.

Thank you for your consideration.

**Annex II: Amharic version questionnaire**

ሰላም ነዎት፡- ስሜ _______ይባላል፡፡ አሁን ከእርስዎ ጋር የተገናኘሁት አድስ አበባ ከተማ ዉስጥ በሚገኙ የመንግስት ሆስፒታሎች ላይ አተኩሮ በሚሰራው ጥናት ላይ መረጃ ሰብሳቢ ሆኜ ነው፡፡ ጥናቱ የሚያተኩረው ከፍተኛ እንክብካቤ ክፍል ዉስጥ ያሉ የጨቅላ(አራስ) ሕፃናት ሞት እና ተዛማጅ ምክኒያቶች ላይ ነዉ፡፡ መረጃዉን የሚንሰበስበዉ በሀዋሳ ዩኒቨርሲቲ ህክምናና ጤና ሳይንስ ኮሌጅ ህ/ብ ጤና ት/ቤት የ Epidemiology ድህረ ምረቃ ተማሪ የሆነዉ እራአን ሽግኝ ለሚሰረዉ ጥናት ነዉ፡፡ ስለሆነም አርስዎ የጥናቱ ተሳታፊ ሆነዉ ስለተመረጡ ስለጥናቱ የተወሰነ ማብራሪያ ስለምሰጥዎት ትኩረትዎን ሰጥተው በጥሞና እንድከታተሉ በትህትና እጠይቃለሁ፡፡

የመረጃው ደህንነት

መጠይቁ የተሳታፊዎችን ሚስጥር ለመጠበቅ ሲባል መለያ ቁጥር ይሰጠዋል፡፡ የጥናቱ ተሳታፊ እናት የምትሰጠን መረጃ ምስጥራዊነቱ የተጠበቀ ነው፡፡ ማንኛውም ዓይነት ስለ እናትየው ማንነት ልገልጽ የሚችል መረጃ በመጠይቁ አይጻፍም፡፡የጥናቱ ውጤት እንዳጠቃሊይ ጥናት እንድወከላቸው ህዝቦች ይወሰዲል እንጂ እንደግለሰብ ለጥናቱ ተሳታፊ ብቻ አይወሰድም፡፡በመጠይቁ ላይም የተሳታፊ ስም አይኖርም፡፡

ጥናቱ በእርስዎ ሙሉ ፈቃደኝነት ላይ የተመሰረተ ነው፡፡ በጥናቱ የመሳተፍም ያለመሳተፍም ሙሉ መብት አለዎት፡፡ የጥናቱ አካል ለመሆን ፈቃደኛ ከሆኑ መልስ ለመስጠት ፍቃደኛ ያልሆኑባቸውን ጥያቄ ላለመመለስ እንድሁም ከጥናቱ በማንኛውም ጊዜ የማቋረጥ ሙሉ መብት እንዳለዎት ላሳስብዎት እፈልጋለሁ፡፡ ከእርስዎ የመሰረታዊ መረጃ ከሰበሰብን በኋላ ለ7 ቀናት የሕፃንዎትን ሁኔታ እንከታትላለን፡፡ ወደ ቤትዎ እንድትመለሱ ከተናገርዎት እድሜዉ ሰባት ቀን እስኪሞላ ድረስ በየቀኑ እንደውልልዎታለን!

ከሊይ በተደረገልዎት ገለፃ መሰረት በጥናቱ ውስጥ ለመሳተፍ ፍቃደኛ ኖት?

ሀ. አዎ-------------- ከሆነ መጠይቁን ይቀጥላል

ለ. አይደለሁም -------- ከሆነ አመሰግናለዉ

**ክፍል-1፡ የሥነ- ህዝብ ፤ ማህበራዊ እና ኢኮኖሚያዊ ጉዳይዎችን በተመለከተ የተዘጋጁ ጥያቄዎች**

| ተ.ቁ | Variables | ኮድ | መዝለል/Skip |
| --- | --- | --- | --- |
| 101 | የሕፃኑ/ ዋ ዕድሜ በመግቢያ ቀን | _____________ |  |
| 102 | የሕፃኑ ጾታ | - 1. ወንድ 2. ሴት |  |
| 103 | የሕፃኑ ክብደት በመግቢያው ቀን |  |  |
| 104 | የእናት ዕድሜ |  |  |
| 105 | የእናት ጋብቻ ሁኔታ | 1. ያላገባች  2. ያገባች  3. የፈተች  4. ባሏ የሞተባት |  |
| 106 | ሀይማኖት | 1. ኦርቶዶክስ  2. ሙስሊም  3. ፕሮቴስታንት  4 . ሌሎች |  |
| 107 | የእናት ትምህርት ደረጃ | 1. ማንበብ እና መጻፍ የማትችል  2. ማንበብ እና መጻፍ የምትችል  3. 1 ኛ -8 ኛ ክፍል የተማረች  4. ከ 9 ኛ -12 ኛ ክፍል የተማረች  5. ኮሌጅ እና ከዚያ በላይ የተማረች |  |
| 108 | የአባት ትምህርት ደረጃ | 1. ማንበብ እና መጻፍ የማትችል  2. ማንበብ እና መጻፍ የምትችል  3. 1 ኛ -8 ኛ ክፍል የተማረች  4. ከ 9 ኛ -12 ኛ ክፍል የተማረች  5. ኮሌጅ እና ከዚያ በላይ የተማረች |  |
| 109 | የእናት ሥራ ሁኔታ | 1. የቤት እመቤት  2. የግል ሥራ  3. አርሶ አደር  4. ነጋዴ  5. ሌሎች ( ይግለጹ) |  |
| 110 | የቤተሰብ ብዛት | __________ ( በቁጥር) |  |
| 111 | የወር ገቢ | _______________ብር |  |
| 112 | የመኖሪያ ቦታ | 1. ከተማ 2. ገጠር |  |
| 113 | የአልኮል መጠጥ የመጠጣት ልማድ አለዎት? | 1. አዎ 2.አይደለም |  |
| 114 | ጫት የመቃም ልምድ አለዎት? | 1. አዎ 2.አይደለም |  |

**ክፍል ሁለት፡ከእርግዝናና ወሊድ እንዲሁም የእናት ጤና አገልግሎት ሁኔታ ጋር የተያያዙ ጥያቄዎች**

| ተ.ቁ | Variables | Code /ኮድ | Skip/ዝለል |
| --- | --- | --- | --- |
| 201 | ይህንን ህጻን እርጉዝ ሆነዉ የቅድመ ወሊድ ክትትል አድርገዉ ነበር? | 1. አዎ  2. አይደልም | 2 ከሆነ  ወደ 203 ይሂዱ |
| 202 | ስንት ግዜ ለክትትል ሄደዉ ነበር? | 1. አንድ ጊዜ  2. ሁለት ጊዜ  3. ሶስት ጊዜ  4. አራት ጊዜ |  |
| 203 | ከዚኛዉ ህጻን እርግዝና በፊት አርግዘዉ ያዉቃሉ? | 1. አዎ  2. አይደለም | 2 ከሆነ  ወደ 207 ይሂዱ |
| 204 | በአጠቃላይ ስንት ግዜ አርግዘዋል ( የእርግዝና ብዛት) | ___________ |  |
| 205 | በህይወት የተወለዱ ልጆች ብዛት ( ፓሪቲ) | ___________ |  |
| 206 | ከ አሁኑ ወሊድ እና ከቀድሞ / ከበፊቱ/ ወሊድ መካከል ምን ያህል ግዜ ነበር? | _______ በ ዓመት |  |
| 207 | የአሁኑን ልጅ የት ነበር የወለዱት? | 1.ጤና ተቋም 2.ቤት |  |
| 208 | በአሁኑ እርግዝና ስንት ልጅ ነበር የወለዱት | 1. አንድ  2. መንታ  3. ሌላ ቁጥር( ግለጽ) |  |
| 209 | የተወለደው በማን ነው? | 1. በዘመድ  2. በባሀላዊ አወላጅ  3. በጤና ኤክስቴንሽን ሰራተኛ  4. በጤና ባለሙያ  5. ሌላ(ግለጨጭ) |  |
| 210 | በምንድ ነበር የወለዱት? | 1. በምጥ  2. በመሳሪያ ታግዘው  3. በኦፐርሽን[በቀዶጥገና] |  |
| 211 | በእርግዝና ወቅት ደም ግፊት ነበረብዎት ? | 1. አዎ 2. አይደለም |  |
| 212 | በእርግዝና ወቅት ደም የመፍሰስ ችግር አጋጥምዎት ነበር? | 1. አዎ 2. አይደለም |  |
| 213 | በእርግዝና ወቅት የአባለዘር በሽታ ነበረብዎት? | 1. አዎ 2. አይደለም |  |
| 214 | በእርግዝና ወቅት የሽንት ቧንቧ እንፈክሽን ነበረብዎት? | 1. አዎ 2. የለም |  |
| 215 | በወሊድ ግዜ ትኩሳት ነበረብዎት? | 1. አዎ 2. የለም |  |
| 216 | ከዚ በፊት ዉርጃ ፈጽመዉ ያዉቃሉ? | 1. አዎ 2. አይደለም | 2 ከሆነ ወደ 218 ሂድ |
| 217 | ምን አይነት ውርጃ ነበር ያጋጠምዎት? | 1. በራሱ ግዜ 2. ኣስወርጄ |  |
| 218 | የአሁኑ ሕፃን በስንት ወሩ ነበር የተወለደው？ | ( በ__ሳምንት) |  |
| 219 | የኧች አይ ቪ ምርመራ ዉጤትዎት ምን ነበር？ | 1.ፖዘቲቭ  2. ነጋቲቭ  3.ለላ (ግለጭ) |  |
| 220 | በህክምና የተረጋገጠ የሱካር በሽታ አለብዎት? | 1. አዎ  2. የለብኝም |  |
| 221 | የእናትየዉ ሄሞግሎቢን መጠን? | __________ |  |
| 222 | ከዚህ በፊት ከተወለደ በኋላ ልጅ ሞቶብዎት ያዉቃል? | 1. አዎ 2. አይደለም |  |
| 223 | ሌላ በዚ እርግዝና ጊዜ /ወሊድ ላይ/ የጋጠምዎት ችግር ነበር? ይግለጹ | ___________ |  |
| 224 | ከየት ነበር ወደዚ ያመጡት | 1. ከዘህ ሆስፕታል  2. ከሌላ ጤና ተቋም  3. ከቤት |  |

**ክፍል ሶስት፡ ጨቅላ/አራስ/ እፃናት ገር ተያይዘዉ ያሉ ችግሮችን መጠየቅ**

| **ተ.ቁ** | **Variable** | **ኮድ** | **ዝለል/skip** |
| --- | --- | --- | --- |
| 301 | ህፃኑ ኢንደተወለደ አልቅሶ ነበር？ | 1. አዎ 2 አይደለም |  |
| 302 | የመጀመሪያ ደቂቃ APGAR ነጥብ ስንት ነበር？ | **_____________** |  |
| 303 | የ5ኛ ደቂቃ APGAR ነጥብ ስንት ነበር? | **__________** |  |
| 304 | መግብያ ላይ የነበረዉ የህጻኑ ሙቀት በ °C |  |  |
| 305 | ህፃኑ ስወለድ ሪሰስቴት ተደርጎ ነበር? | - 1. አዎ 2. አይ |  |
| 306 | በተወለደዉ በአንድ ሰዓት ዉስጥ ካንጋሮ እናት እንክብካቤ (KMC)ተደርጎ/ጋ ነበር**？** | 1. አዎ  2 አይ |  |
| 307 | ህጻኑ የእናት ጡት ማጥባት ጀምረዋል? | 1. አዎ 2. አይ | 2 ከሆነ  ወዳ 309ሂድ |
| 308 | መች ነዉ የእናት ጡት ማጥባት የጀምረዉ? | - 1. ተወልዶ አንድ ሰዓት ዉስጥ  1. ተወልዶ ከ አንድ ሰዓት ቧላ |  |
| 309 | በዘህ ሰባት ቀናት ዉስጥ የጨቅላ እፃኑ ምግብ ምን ነበር? | - 1. የእናት ጡት ወተት ብቻ  1. ከሌላ ተጨማሪ ምግብ ጋር 2. ሌላ (ግለፅ) |  |
| 310 | አራስ እፃኑ ሴፕስስ(እንፌክሽን በደሙ ዉስጥ) አለዉ/አላት? | 1. አዎ 2. አይ ደለም |  |
| 311 | አራሱ እፃን የመተንፈሻ አካላት ላይ ከፍተኛ የአተነፋፈስ ችግር（RDS) ምልክቶች አለዉ/አላት? | 1. አዎ  2. አይደለም |  |
| 312 | የህጻኑ የመታፈን ችግር (አስፊክሲያ) አለዉ/አላት? | 1. አዎ 2. አይ ደለም |  |
| 313 | ህጻኑ ጃዉንድስ (ወይቦ) አለዉ/አላት? | 1. አዎ 2 . አይ |  |
| 314 | አራስ ሕፃኑ ሲወለድ የተፈጥሮ ሰውነት እክል ነበረው? | 1. አዎ  2. አይ | 2 ከሆነ  ወዳ 316 ሂድ |
| 315 | ካለ ምን አይነት እክል ነው(ይግለጹ) |  |  |
| 316 | አራስ ሕፃኑ ሌላ ችግር ካለው/ላት ይግለጹ |  |  |

• የተወለደበት/ችበት ቀን ____________/__________/____________

• የመግቢያ ቀን - - - - - - - - - - - - - - / - - - - - - - - - - - - - - - - - - - - - - - - ----

• ሕፃኑ የሞተበት/ ችበት ቀን - - - - - - - - - - - / - - - - - - - - - - / - - - - - - - - - - - - - - -

• ሕፃኑ ከህክምናው/ ዋ የጠፈበት/ ችበት ቀን _________ / _______ ________

• ሕፃኑ ከክትትል ያቋረጠበት/ችበት ቀን - - - - - - - - - - - / - - - - - - - - - - / - - - - - - - -

• ሕፃኑ ወደ ሌላ ህክምና የተዛወረበት/ችበት ቀን _________ / _________ ______

• ሕፃኑ ክትትል የተደረገበት ጠቅላላ ቀናት ብዛት___________ ፡፡

ጥናቱ ያለቀበት ቀን ______________________
